# The Proactive Shift in Managing an Older  Workforce 2009–2017: A Latent Class Analysis of Organizational Policies

**DOI:** 10.1093/geront/gnaa037

**Published:** 2020-05-04

**Authors:** Konrad Turek, Jaap Oude Mulders, Kène Henkens

**Affiliations:** 1 Netherlands Interdisciplinary Demographic Institute (NIDI-KNAW & University of Groningen), The Hague, The Netherlands; 2 Department of Sociology, University of Amsterdam, The Netherlands; 3 University Medical Center Groningen (UMCG), The Netherlands

**Keywords:** Older workers, Labor market, Employers, Human resource policy

## Abstract

**Background and Objectives:**

Longitudinal perspectives on how organizations react to workforce aging are missing in the literature. In this study, we fill this gap and ask how organizations deal with older workers, how their approaches change over time, and in which sectors of the economy and types of organizations the changes were most profound.

**Research Design and Methods:**

Data come from two large-scale employer surveys: 2009 (*n* = 1,077) and 2017 (*n* = 1,358), representative for the Netherlands. We use a three-step group-comparison latent class analysis combined with a multinomial logistic model.

**Results:**

We found four clusters of organizations based on their practices regarding older workers—those trying to activate and develop their employees (active), focusing solely on exit measures (exit), implementing a combination of development, accommodating and exit measures (all), and practicing no age management (none). We find a major shift in employers’ approaches to aging workforces between 2009 and 2017, with strong decreases in those that offered no age management (47%–30%) and those focusing on exit measures (21%–6%), and an increase in active organizations (19%–52%). Active age management is no longer concentrated in large and developing organizations, but has become a standard human resources tool economy-wide.

**Discussion and Implications:**

Overall, there is a long-term trend away from exit measures toward the application of proactive age management measures. More involvement of employers in retaining older adults in the workplace may signal a growing awareness of the changing demographic reality.

Due to demographic changes, labor markets in developed countries are aging. Threats of diminishing labor supplies and increasing costs of pension systems have forced governments to implement policies to promote longer working lives. It is often argued that these changes force organizations to adjust their policies and practices to a more inclusive approach toward older workers (Henkens et al., 2018; Silverstein, 2008; Walker, 2005). However, many earlier studies suggest that organizations across countries are more inclined to send older staff members to early retirement than to retain them (Harper, Khan, Saxena, & Leeson, 2006; Van Dalen, Henkens, & Schippers, 2009). In recent years, there have been signals that employers are more aware of the challenges of an aging workforce (SHRM, 2014) and gradually change their policies and practices to support longer working lives (Moen, Kojola, & Schaefers, 2017). However, a large-scale, longitudinal perspective on how organizations have reacted to demographic challenges is missing in the literature.

An increasing number of studies on organizational approaches toward aging workforces has provided insights into different types of age management (e.g., Kooij, Jansen, Dikkers, & De Lange, 2014; Kooij & Voorde, 2015; Van Dalen, Henkens, & Wang, 2015), but these studies lack a comprehensive view on how organizations approach the aging workforce generally. For example, whether particular approaches are concentrated in some segments of the economy, or whether there are specific groups of organizations that implement different types of approaches simultaneously, are unknown. Existing studies also largely ignore the important group of organizations that offer no specific human resources (HR) practices for older workers. Research also offers limited evidence on the evolution of organizational policies. Panel surveys repeated on the same sample of organizations are missing in the field, and longitudinal perspectives of cross-sectional research repeated across time are scarce (Conen, Henkens, & Schippers, 2011).

We fill this gap by asking how organizations approach older workers, how these approaches change over time, and in which sectors of the economy and which types of organizations changes were most profound. Data come from two large-scale employer surveys from 2009 and 2017, representative for the Netherlands, a country with one of the highest increases in age at labor force exit in Europe (OECD, 2019).

This study contributes to the literature in three ways. First, to obtain a comprehensive view of organizational approaches toward older workers, we use latent class analysis (LCA) to distinguish clusters of organizations with similar bundles of practices that are implemented simultaneously (Kooij et al., 2014; Oude Mulders, Henkens, & Schippers, 2015). LCA allows us to study whether specific approaches are concentrated in some types of organizations and to distinguish organizations without any specific policies toward older workers. Organizational policies are not predefined on a theoretical basis but are found as the result of an analytical process. Second, a longitudinal perspective allows us to study ways employers respond to changing contexts (Bohlmann, Rudolph, Zacher, & Truxillo, 2018). Third, we study the Netherlands, a country that is at the forefront of implementing policies to stimulate and facilitate longer working lives. Retiring early was common until the early 2000s, but the employment rate for 60–64-year- olds increased from 22% in 2003 to 51% in 2015, and the average age at labor force exit increased from 61 in 2006 to 65 in 2018 (Statistics Netherlands, 2019). This extension of working lives is largely attributable to policy reforms, such as limiting early retirement opportunities in 2006 and gradually increasing the pension age from 65 in 2013 to 67 in 2024 (Sonnet, Olsen, & Manfredi, 2014). Although the changes were largely successful from a policy perspective, there has been backlash from both employees, who feel forced to continue working (Van Solinge & Henkens, 2017), and employers, who feel forced to continue employing older workers due to high levels of employment protection legislation (Van Dalen, Henkens, & Oude Mulders 2019). Other relevant characteristics of the Dutch labor market and pension system are automatic enrollment in a pension fund and mandatory retirement at state pension age for the large majority of employees. In comparison with other EU and OECD countries, Dutch organizations provide on average more opportunities to learn and more supportive management, and also use the skills of their workers more efficiently (OECD, 2017).

## Organizational Policies for Older Workers

### Types of Approaches and Practices

Human capital theory provides an economic framework for analyzing organizational policies for older workers. Derived from analyses of relationships between wages and marginal productivity across careers, classic human capital theory assumes that these two factors correlate closely, and that the degree of an individual’s general and specific human capital determines marginal productivity (Mincer, 1964). The fundamental production factors for both types of human capital are continuous education and experience. Although individual experience accumulates during the career, investments in education are expected to decrease in older ages because of the proximity of retirement and lower expected returns from investment (Hutchens, 1988). According to human capital theory, the last stage of a career is therefore characterized by a drop in human capital and lower productivity. To balance costs and benefits—from a traditional neoclassical perspective—remuneration should decline accordingly. Many studies from 1950 to 1970 suggested, however, that wage declines are rare among older workers (Polachek & Siebert, 1993). Lazear (1979) was among the first to address this issue; his deferred payment model explains the gap between wages and productivity as the result of a long-term contract between employee and employer, where late-career overpayments balance earlier underpayments and thus motivate workers to stay with an organization. A functional consequence of this model is retirement, which is necessary when long-term costs exceed benefits. From a human capital perspective, organizations that want to avoid such costly gaps can either cut costs by pushing older workers out of employment or invest in human capital development. Most economic studies focus on the former, treating age-related decreases in productivity as more difficult to handle (Hutchens, 1988).

Numerous studies of employers’ attitudes and organizational approaches accord with the human capital theory: organizations are reluctant to train older workers, and the early-exit policy has been the most common, accepted, and easiest solution for dealing with older employees (Conen et al., 2011; Kooij, Jansen, Dikkers, & De Lange, 2010; Van Dalen et al., 2009, 2015). Exit policies comprise measures that enable earlier or some form of gradual retirement. Early retirement schemes are in principle defined by the pension system, but employers might encourage older workers to take this option by, for example, offering additional benefits or providing administrative support. Gradual retirement refers to a situation in which a worker engages in paid employment on a limited scale during the period between full-time work and full retirement. A simple reduction of work hours at the same job is sometimes called phased retirement. More attention has been paid to partial retirement, often referred to as bridge employment, which means changing to a different job that has fewer hours (Beehr & Bennett, 2014). A route into a bridge job often leads through early retirement, and employers can force such solutions to optimize employment costs and provide more flexibility.

Psychology and management studies criticize some elements of human capital theory as being too general and simplistic. Contemporary approaches refrain from treating individual productivity as the most important worker attribute, and instead either focus on productivity measured at the organizational level or consider aspects of individual performance (Ng & Feldman, 2008). The relationship between productivity and age is not necessarily negative, with strong evidence suggesting it is zero, on average, and diverse across job types (Sturman, 2003). For example, Warr (1993) distinguished between jobs in which older workers’ productivity usually increases (e.g., craftsmen or HR managers), decreases (e.g., physical workers, high IT specialists), or stays unchanged (routine jobs, scientists) depending on physical and cognitive requirements and the role of experience. Better management and organization of work can additionally increase individual performance at older ages in most of the jobs (Silverstein, 2008). The HR management perspective also criticized deferred payment models (Lazear, 1979) for overestimating the role of expected timing of retirement when accounting for investment return. Younger workers often have greater propensities to leave an organization unexpectedly, whereas retirement of older workers is a more predictable event, and distributions of employer-sponsored training often depart from what the human capital model predicts (Gielen & Van Ours, 2006).

These findings point to HR management as essential to understanding and shaping employment at older ages. Contrary to a cost reduction, human capital approach, HR management models suggest proactive ways of dealing with potential wage–productivity gaps. Two popular and complementary models address the idea behind age management well. The Job Demands–Resources (JDR) model focuses on adjustments to job demands, job environments, and individual resources to enhance employee performance (Kooij et al., 2010, 2014), and the Ability Motivation and Opportunities (AMO) model suggests that work at older ages can be facilitated by HR practices that increase employee abilities, stimulate motivation, and provide opportunities to perform (Bal, Kooij, & De Jong, 2013). In line with the JDR and AMO models, two essential approaches can support prolonged productive employment of older workers—development and accommodation.

The goal of developmental HR is to increase workers’ functioning, with employer-provided training as its core element (Hansson, 2008). A common argument is that continuous acquisition and adjustment of workers’ skills is as important to extending working lives (Picchio & van Ours, 2013) as improving an organization’s productivity and competitiveness (Barabasch, Dehmel, & Loo, 2012). Innovations and technological progress require continuous learning to maintain productivity and should encourage investments in the development of older workers. However, as mentioned above, evidence suggests that older people are offered fewer opportunities for training. Rationality of employers’ decisions regarding investments in human capital is limited by their knowledge and assumptions about workers’ potential and attitudes (Bishop, 1987). Most of all, prevailing negative stereotypes of older workers regarding their lower willingness and ability to learn are root causes of these discriminatory practices (Posthuma & Campion, 2009).

Accommodation policies, in line with the JDR and AMO models, address the problem of declines to capacities related to aging by adjustment of work environments (Kooij et al., 2014). Employers often have worries about the loss of productivity at older ages due to decreasing health and absenteeism at work (Böckerman & Ilmakunnas, 2008). Although older workers, also in the Netherlands (Vanajan, Bültmann, & Henkens, 2019), often experience health conditions that limit their work abilities, appropriate accommodative measures may support them. Some measures might allow working at lower levels (e.g., reducing physical requirements), maintaining the current level of functioning (e.g., flexible arrangements), or helping workers use their potential (e.g., changing tasks). The most popular and important accommodative measures include flexible working arrangements and implementing ergonomic measures (Conen et al., 2011). Flexible working times allow employees to codecide when they start and end their work days, helping employees to balance work with leisure activities, social connections, and care obligations. They also facilitate positive work-related attitudes in older age and might attract older individuals to an organization (Rau & Adams, 2005). Some research suggests a relationship between flexible working arrangements and both higher individual performance (Kelly & Moen, 2007) or firm productivity (De Menezes & Kelliher, 2011). The purpose of ergonomics is to redesign physical and technological aspects of jobs and work environments to support health, wellbeing, safety, and productivity in older workers (Truxillo, Cadiz, Rineer, Zaniboni, & Fraccaroli, 2012). Many physical capabilities, including strength, agility, speed of movement, and motor skills, decline with age, affecting the ability to work and increasing work-related risks and stress. The literature discusses ergonomic interventions that compensate for such declines, such as improvements to seating designs, worksite illumination, and safety equipment, and reductions to lifting, carrying, repetitive tasks, and background noise (Roper & Yeh, 2007).

### Factors That Affect Age Management Approaches

Organizational research suggests that the type and scope of age-related policies depend consistently on several organizational characteristics. Larger organizations tend to implement more measures of different types, mainly due to their need for more diverse HR management of a large number of workers (Conen et al., 2011; Moen et al., 2017). Organizations with higher shares of older employees focus mainly on facilitating early exit rather than accommodation or development, which reflects the fact that retention is not a priority for organizations (Conen et al., 2011; Fleischmann, Koster, & Schippers, 2015; Van Dalen et al., 2009, 2015). However, organizations that experience staff shortages are more likely to adapt accommodation and development approaches as a solution for recruitment problems (Van Dalen et al., 2015). Studies also show that labor unions influence personnel policies by stimulating development of accommodative procedures that aim to improve person–job fit, and implementation of exit or early retirement schemes, especially in the face of mass layoffs (Taylor, McLoughlin, Brooke, Di Biase, & Steinberg, 2012; Fleischmann et al., 2015; Van Dalen et al., 2015). Organizations in knowledge-intensive and high-skill sectors have higher demand for skills and invest more in development, though not necessarily in older workers (Fleischmann et al., 2015; Van Dalen et al., 2015). In our goal to analyze how organizational approaches toward older workers changed over time and where the changes were most significant, we will control for the factors described above. We consider both changes in the distribution of organizational characteristics over time, as well as changes of their role for implementation of age management strategies.

## Methods

### Data

Data came from two large-scale cross-sectional surveys conducted among Dutch employers in 2009 (*n* = 1,077) and 2017 (*n* = 1,358). Both surveys were similar in design and focused on personnel policies, HR management, and employment practices regarding the extension of working lives. For both surveys, a random sample of organizations with at least 10 employees was drawn from a sampling frame including all registered organizations in the Netherlands (branches of the same organization were not considered). Samples were stratified by size and sector, to ensure a sufficient number of responses from large organizations and organizations from the public sector (which would otherwise be underrepresented). Poststratification weights were used to correct for sample stratification, so that results are representative for the population of Dutch organizations with more than 10 employees. In both years, surveys were sent by post, followed by two reminders to participate several weeks apart. The 2017 survey additionally featured an online response option, which yielded half of the 2017 responses. Overall, response rates were the same for both surveys at 23%, which is similar to other surveys among organizations in the Netherlands (Conen et al., 2011) and other countries (Baruch & Holtom, 2008). The surveys were completed by CEOs, owners, or directors (2009: 41%; 2017: 47%), HR managers (34%; 27%), HR employees (4%; 12%), general managers (10%; 6%), and other employees (11%; 8%).

### Measures

#### Dependent Variables

Application of policies that address older workers (employees over the age of 50) was measured using a list of items that was based on extant research on age-conscious personnel policies and applied in other studies (Van Dalen et al., 2009, 2015). During both waves, employers were asked, “Are the following measures regarding older workers currently implemented in your establishment?” (1 if applied; 0 if not), for six measures—ergonomic measures, training, flexible work hours, part-time retirement, gradual retirement, and early retirement (Table 1). These items represent a mixture of HR practices concerning development, accommodation, and exit.

**Table 1.** Characteristics of Organizations and Wording for Variables Used in the Analysis (in percentages)

	2009	2017	Significance of change	Wording
Sector (%)				“Within which of the following industry sectors does your establishment operate?” 18 industries reduced to three main sectors, according to the NACE 2.0 (Statistical Classification of Economic Activities in the European Community).
Industrial	24.7	29.2		
Services	59.0	57.4		
Public	16.3	13.4		
Size (%)				“Approximately how many people are currently employed in this establishment?”
10–49	79.9	76.7		
50–249	16.0	18.1		
250+	4.1	5.2		
Strong role of labor unions (%)	16.2	15.1		“The influence of labor unions on personnel policies is clearly visible in this establishment” (0 = no; 1 = yes)
Knowledge-intensive (%)	68.6	72.5		“The knowledge intensity in our establishment is high” (0 = no; 1 = yes)
Requires regular training (%)	59.0	50.7	*	“Working in our establishment requires regular additional training” (0 = no; 1 = yes)
Experienced shortages (%)	48.3	69.3	***	“Has your organization recently experienced difficulties in finding staff?” (0 = no; 1 = yes)
Share of workers 50+ (%)				“What percentage of employees is 50 years or older?”
0–9	26.97	12.07	***	
10–19	22.59	22.06	***	
20–29	22.49	19.47	***	
30–39	10.47	16.57	***	
40–49	8.97	12.05	***	
50–59	5.43	9.36	***	
60–100	3.09	8.42	***	
(average)	20.4	28.8	***	
Share of women (%)				“What percentage of employees is female?”
0–9	18.05	19.48		
10–19	22.07	20.34		
20–39	16.2	18.85		
40–59	19.64	17.31		
60–79	12.53	13.14		
80–100	11.51	10.89		
(average)	37.0	35.1		
Practices toward older workers (%)				“Are the following measures regarding older workers currently implemented in your establishment?” (0 = no; 1 = yes)
Ergonomic measures	28.0	50.4	***	
Training older	8.1	41.5	***	
Flexible hours	31.5	55.1	***	
Part-time retirement	28.5	12.8	***	
Gradual retirement	20.4	20.1		
Early retirement	31.8	14.9	***	
*N*	1,077	1,358		

*Note*: Significance of the differences between years: *p < .05; **p < .01; ***p < .001.

#### Control Variables

We controlled for several organizational characteristics during multinomial logistic regression analysis, including size, sector, percentage of female employees, workers 50 years and older, knowledge and training intensity, experiencing recruitment problems, and the influence of unions on personnel policies (Table 1).

### Missing Values

In a pooled sample of 2,435 organizations, there were 4.3% observations with missing values for all items on application of policies, which were consequently excluded, leaving 2,331 cases for analysis. In the final sample, 186 cases (8%; 40 in 2009 and 146 in 2017) had at least one missing value in covariates of the regression model (between 0.4% for organizational size and 4.3% for recruitment problems). These missing values were imputed iteratively using multivariate imputation with chained equations (MICE) with additional auxiliary predictors, conditioned on year (50 imputations) and based on the full sample. Imputation and analyses were performed using Stata 15.

### Analytical Approach

We used a three-step approach to LCA. During the first step, we estimated the unconditional group-comparison LCA model (grouped by wave) using only indicators of class membership (items on application of policies). As the literature recommends, we did not include predictors of membership at the same stage as the classification model to provide more stable categorization (Asparouhov & Muthén, 2014). The group-comparison model was specified so that LCA was conducted on the pooled sample, constraining measurement to be equal in both waves yet allowing for the constant, related to the size of the cluster, to vary by year. This approach allowed us to compare the distribution of clusters between years, ensuring they were measured similarly. We estimated several models using a different number of clusters, and the decision of which solution was best was based on fit measures and theoretical interpretation (Collins & Lanza, 2010; Tofighi & Enders, 2008). During the second step, we used a standard approach to determine each subject’s most likely class membership by predicting membership based on the highest probability for all clusters, resulting in a categorical variable. An alternative and commonly recommended approach is crisp membership association, which simultaneously models probabilities of membership in all classes as multiple dependent variables (Asparouhov & Muthén, 2014; Vermunt, 2010). However, for a group-comparison model, the categorical approach is the optimal solution because it allows comparison of the effects of predictors and changes between years. The classification accuracy of the model was satisfactory (i.e., entropy > 0.7), and the structure of clusters by year using the crisp and categorical approach was similar (Supplementary Figure A1). During the third step, we included covariates to predict the most likely class membership using multinomial logistic regression analysis. A model was estimated separately for each wave as a group-comparison model, allowing coefficients and error variances to differ by group but with a correction of standard errors which allows to compare coefficients. To estimate whether the distribution of classes changed between 2009 and 2017 in relation to predictors, we compared predicted probabilities of most likely class membership for pooled-mean values of predictors (Long & Mustillo, 2018). In all tables, we indicate statistical significance of model parameters based on the *p*-values.

## Results

### Identification of Clusters

Table 1 shows descriptive information on how many organizations had applied HR practices that address older workers. Between 2009 and 2017, the popularity of most measures changed significantly. Application of proactive practices increased greatly, with the largest change being training of older workers (from 8.1% to 41.5%) and a nearly doubling of ergonomic measures and flexible work hours. Part-time and early retirement practices decreased by more than half, and gradual retirement remained at the same level.

These HR practices served as indicators for the LCA model. Table 2 shows fit statistics for the estimated models with up to six latent classes. To select the optimal solution, we used statistical and theoretical criteria. The most commonly used measures, Bayesian Information Criterion (BIC) and sample size-adjusted BIC (SABIC), were lowest for the four-cluster solution (Tofighi & Enders, 2008). Entropy (0–1) was calculated based on posterior probabilities of membership, with larger values suggesting better latent class separation (Collins & Lanza, 2010: 75). All models demonstrated a similar level of entropy, between 0.7 and 0.78. The Bootstrapped Likelihood Ratio Test (BLRT) suggested no increase to fit between four- and five-class solutions (Nylund, Asparouhov, & Muthén, 2007). We considered the number of small (below 10% of organizations in a given year) and very small (below 5%) clusters, which can hinder interpretation. The first small cluster appeared in the four-class solution. Based on fit criteria, parsimony, and interpretability, the four-class model offered the best representation of data.

**Table 2.** Model Fit Evaluation Information for Six Different LCA Models of Organizational HR Practices in 2009 and 2017

Number of classes	LL	*df*	BIC	SABIC	Entropy	BLRT *p*-value	Clusters <5%	Clusters <10%
2	−7,493.04	13	15,086.9	15,045.6	0.777	—	0	0
3	−7,269.87	20	14,694.8	14,631.3	0.746	.000	0	0
4	−7,221.81	27	14,653.0	14,567.2	0.702	.000	0	1
5	−7,211.22	34	14,686.1	14,578.1	0.713	.280	1	2
6	−7,205.97	41	14,729.8	14,599.6	0.769	.810	2	4

*Notes:* The table shows LL (Log-Likelihood) and *df* (degrees of freedom) for particular solution. Low values of BIC (Bayesian Information Criterion) and SABIC (sample size-adjusted BIC) indicate a better fit. Entropy (0–1) is calculated based on posterior probabilities of membership; larger values suggest better class separation. *p*-value for BLRT (Bootstrapped Likelihood Ratio Test) shows difference in fit between the k-classes model and a k – 1 classes model; significant results suggested improvement (test performed on unweighted data in Mplus 8.3). Clusters indicate number of clusters with estimated size lower than 5% or 10% in a given year. HR = human resource; LCA = latent class analysis.

Table 3 shows item–response profiles for the four-class model, with values representing the average probability that organizations that belonged to that class applied particular HR practices. The first cluster of organizations was characterized by no specific practices regarding older workers (i.e., “none”). The item–response probability was close to zero, except flexible work hours (0.24), which was one of the most popular practices during both waves. The second cluster included organizations that focused solely on exit practices (i.e., “exit”), with the strongest coefficients for part-time (0.60) and early retirement (0.80). Gradual retirement was estimated to have been applied in 43% of organizations in this class. The third class was labeled “active” because it was characterized by a combination of accommodation (i.e., ergonomic measures and flexible hours, with probability over 0.6) and development (i.e., training, with probability 0.48). The last group was characterized by diverse policy strategies, with application of all practices with high probability (i.e., “all”).

**Table 3.** Latent Classes Item–Response Profile for the Four-Class Model of Organizational HR Policies (values indicate the average probability that organizations from the class applied particular HR practices)

Indicator	Clusters			
	None	Exit	All	Active
Ergonomic measures	0.02	0.30	0.82	0.68
Training older	0.01	0.04	0.61	0.48
Flexible hours	0.24	0.26	0.65	0.64
Part-time retirement	0.03	0.60	0.74	0.05
Gradual retirement	0.02	0.43	0.76	0.12
Early retirement	0.00	0.80	0.85	0.05

*Note*: HR = human resource.

### Changes in Class Prevalence

**Figure 1.**
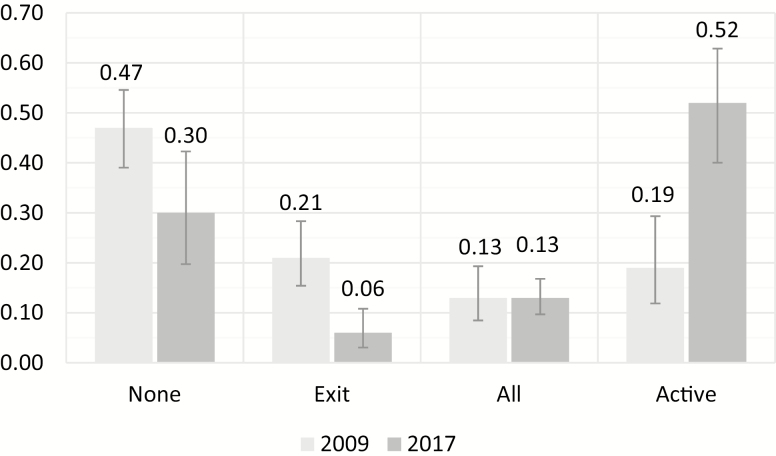
Share of four latent classes distinguished based on organizational Human Resources practices in 2009 and 2017 (in percentages).

The four clusters were identified for both waves in the same way by imposing equal measurement, but the parameter representing their prevalence was unconstrained, which allowed us to investigate whether cluster sizes changed between 2009 and 2017 (Figure 1). In 2009, 47% of Dutch organizations belonged to the *none* cluster, but the group significantly decreased (*p* < .001) to 30% by 2017. A significant reduction was also observed for the *exit* class, from 21% to 6%. The share of *active* organizations more than doubled over the period, from 19% to 52%, and the prevalence of the *all* cluster remained the same at 13%.

Besides population-level changes, we are also interested in what happened across sectors and within types of organizations. To address this question, we conducted multinomial logistic regression analyses on the four-category variable, separately for each wave, indicating most likely class membership. Model coefficients appear in Supplementary Table A1, yet due to their complexity, results of a multinomial model are more accessible in the form of predicted probabilities for cluster membership, shown in Table 4 (Long & Mustillo, 2018). Estimates were for pooled-mean values of predictors. Given the model’s nonlinearity, other reference values produced different predictions, and thus, the row with *Total* values differs from Figure 1 but led to similar conclusions regarding relative differences between predictors.

**Table 4.** Average Probability of Membership in Four Latent Classes Distinguished Based on Organizational HR Practices in 2009 and 2017 and Change Over Time

1		None			Exit			All			Active		
		2009	2017	Change	2009	2017	Change	2009	2017	Change	2009	2017	Change
	2	3	4	5	6	7	8	9	10	11	12	13	14
Sector	Industrial	0.41	0.34	−0.07	0.22	0.05	−0.18***	0.08	0.11	0.03	0.13	0.37	0.24***
	Services	0.60	0.32	−0.27***	0.11	0.01	−0.10***	0.07	0.05	−0.03	0.12	0.54	0.43***
	Public	0.35	0.33	−0.02	0.25	0.03	−0.22***	0.16	0.04	−0.12**	0.11	0.52	0.40***
Size	1–49	0.60	0.39	−0.22***	0.14	0.02	−0.12***	0.07	0.04	−0.03	0.11	0.50	0.39***
	50–249	0.24	0.18	−0.07	0.27	0.02	−0.25***	0.15	0.19	0.03	0.17	0.49	0.32***
	250+	0.11	0.14	0.03	0.17	0.03	−0.14***	0.46	0.30	−0.15*	0.09	0.35	0.26***
Knowledge intensity	0	0.49	0.46	−0.03	0.16	0.02	−0.14***	0.08	0.07	−0.02	0.14	0.37	0.23***
	1	0.52	0.28	−0.23***	0.15	0.02	−0.14***	0.09	0.06	−0.03	0.12	0.54	0.43***
Requires regular training	0	0.59	0.39	−0.20**	0.16	0.02	−0.14***	0.06	0.04	−0.02	0.09	0.46	0.37***
	1	0.44	0.28	−0.16**	0.16	0.02	−0.14***	0.11	0.08	−0.03	0.15	0.52	0.37***
Experienced shortages	0	0.53	0.37	−0.16**	0.17	0.02	−0.15***	0.09	0.07	−0.02	0.10	0.43	0.33***
	1	0.49	0.31	−0.19***	0.15	0.01	−0.13***	0.08	0.05	−0.03	0.14	0.53	0.39***
Strong role of labor unions	0	0.55	0.35	−0.21***	0.14	0.02	−0.12***	0.08	0.05	−0.03	0.12	0.49	0.37***
	1	0.28	0.25	−0.03	0.28	0.02	−0.26***	0.11	0.09	−0.02	0.13	0.50	0.37***
Perc. older	0–9	0.70	0.56	−0.14	0.08	0.01	−0.07**	0.03	0.04	0.01	0.14	0.34	0.19**
	10–19	0.53	0.38	−0.15	0.15	0.01	−0.14***	0.08	0.03	−0.05	0.16	0.51	0.35***
	20–29	0.44	0.28	−0.16*	0.15	0.03	−0.12***	0.11	0.04	−0.08**	0.17	0.60	0.43***
	30–39	0.48	0.19	−0.29**	0.23	0.04	−0.20**	0.09	0.11	0.01	0.07	0.56	0.49***
	40–49	0.31	0.17	−0.14	0.23	0.04	−0.19**	0.22	0.14	−0.08	0.07	0.53	0.46***
	50–59	0.35	0.39	0.04	0.22	0.01	−0.21**	0.16	0.14	−0.03	0.12	0.39	0.27*
	60–100	0.63	0.30	−0.33*	0.17	0.03	−0.14*	0.08	0.13	0.05	0.07	0.46	0.39***
Perc. women	0–9	0.32	0.31	−0.02	0.34	0.04	−0.30***	0.15	0.09	−0.05	0.07	0.44	0.37***
	10–19	0.45	0.33	−0.12	0.21	0.02	−0.19***	0.08	0.07	−0.02	0.15	0.47	0.32***
	20–39	0.59	0.41	−0.18	0.13	0.01	−0.12***	0.06	0.03	−0.03	0.14	0.48	0.34***
	40–59	0.63	0.32	−0.31**	0.07	0.02	−0.05*	0.10	0.06	−0.04	0.12	0.52	0.40***
	60–79	0.48	0.33	−0.15	0.10	0.02	−0.07*	0.11	0.04	−0.07	0.20	0.52	0.32**
	80–100	0.65	0.27	−0.39***	0.17	0.01	−0.16***	0.05	0.08	0.03	0.08	0.56	0.47***
Total		0.51	0.33	−0.18***	0.16	0.02	−0.14***	0.09	0.06	−0.03	0.12	0.49	0.37***

*Notes:* Based on probabilities predicted from multinomial logistic regression model, including all predictors and with multiple imputation of missing values. Prediction for the pooled-mean values of predictors. Rows contain categories of predictors. There are three columns for each cluster—probability of cluster membership in 2009 and 2017, and changes between 2009 and 2017 expressed in pp. HR = human resource.

*p < .05; **p < .01; *p* < .001.

The rows in Table 4 contain control variables with organizational characteristics for which probabilities were estimated. There are three columns for each cluster—probability of cluster membership in 2009 and 2017, and changes across years expressed in percentage points (pp.). For example, in 2009, the active approach was applied in 12% of organizations in the service sector. In 2017, this share changed to 54%, indicating an increase of 43 pp., which is much higher than the increase in the industrial sector (24 pp.) but similar to that in the public sector (40 pp.). Prevalence of the *none* group decreased in the service sector from nearly 60% to 32% (−27 pp.), but did not change significantly in other sectors.

Exit policies decreased (column 8) and active policies increased (column 14) significantly in all types of organizations. Regarding organizational size, the most proactive shift occurred among small organizations, experiencing a 39 pp. increase to *active* and 22 pp. decrease to *none* approaches. Particularly meaningful is the latter trend, because the share of small organizations with the *none* approach changed from 60% to 39% in comparison to nearly no change in large organizations from 11% to 14%. Knowledge-intensive organizations increased their *active* approaches by 43 pp. in comparison to 23 pp. for non–knowledge-intensive entities, and decreased their *none* clusters by −23 pp. and −3 pp., respectively. There was, however, no such effect for training intensity. Organizations with a strong role of unions experienced a more profound decrease in *exit* strategies (26 pp. vs 12 pp.). Trends for organizations that had experienced shortages were nearly the same as those that did not, and no clear effect from the share of workers aged 50 or over and women in an organization was found.

## Discussion

Although organizations play a strong role in offering employment opportunities to older workers, it was unclear to what extent they adapted age management practices in response to demographic changes and policy reforms in recent years. Using latent class analysis, we distinguish four clusters of organizations based on their practices regarding older workers—those trying to activate and develop their employees (active), focusing on exit measures (exit), implementing all age management practices (all), and practicing no age management (none). The sizes of these clusters changed dramatically between 2009 and 2017, with strong decreases in those that offered no age management (47%–30%) and those focusing on exit measures (21%–6%), and an increase in active organizations (19%–52%).

In comparison to the 2000s, when age management was not a priority for organizations (Conen et al., 2011), we observed a proactive shift, because the *active* cluster comprised more than half of Dutch organizations in 2017. Active organizations used various accommodative and developmental measures to adjust jobs and individual resources in ways that stimulated work in older-age workers. So far, these two types of active policies were largely treated separately (Van Dalen et al., 2015). As this study shows, accommodative practices tend to be implemented alongside developmental measures. The great increase in development practices is striking, as for decades, it was common to assume that employers were reluctant to train older workers. This study suggests that in comparison to 2009, the prevalence of training older workers grew by a factor of five, reaching as much as 41% in organizations by 2017. This change accords with evidence that suggests the Netherlands experienced one of the greatest improvements in older-age training attendance in Europe, with the share of people aged 55–64 who participated in nonformal education in the previous 12 months rising from 36% in 2007 to 51% in 2016 (Adult Education Survey, 2016). Such improvements would be impossible without changes to organizational approaches because opportunities and stimuli provided by organizations are the driver of training in older age (Hansson, 2008). In line with this, the share of Dutch enterprises which provide education activities increased from 75% in 2005 to 85% in 2015 (Continuing Vocational Training Survey, 2015).

Combined with a proactive shift, a strong decline in the prevalence of *exit* policies was also evident, and until recently, they represented primary choices for employers when dealing with older workers (Conen et al., 2011). This is no longer true in the Netherlands, because the cluster reduced to only 6% of organizations. Employers use part-time and early retirement measures less often, partly because policy reforms mean that fiscally attractive early retirement opportunities are no longer available to older workers (Sonnet et al., 2014). Another finding is a cluster of organizations that had no policies for older workers. Extant studies commonly neglected this group, but in 2009, it represented the largest cluster, comprising nearly half of Dutch organizations. It remained substantial in 2017, though reduced to 30%, suggesting that an increasing number of organizations are moving away from their passive approach to workforce aging.

Results suggest that *active* measures are no longer concentrated in large and developing organizations, but have become standard HR tools economy-wide. The greatest shift in proactive age management occurred among small organizations, an important and populous group that so far was least interested in retaining older workers. Small organizations usually have less-developed management structures and less-diverse workforces. Among knowledge-intensive organizations, the cluster without age management was replaced by the *active* approach. Innovative and high-skill sectors have been primarily interested in younger employees, but with tightening labor markets and increased education of next generations, they recognize older workers as worth investing in and retaining (Barabasch et al., 2012). The *exit* approach reduced in organizations with strong labor unions, a departure from the model in which unions opted for exit routes as safe solutions under risk of mass layoffs (Van Dalen et al., 2015).

The proactive shift in organizations’ age-management practices is likely connected to four processes (Cascio, 2019; Henkens et al., 2018; Walker, 2005). First, demographic changes in the labor force became a tangible challenge across the entire economy, and employers reacted accordingly. Faced with increasing numbers of older workers in the workforce, and coupled with the fact that such workers were, on average, more highly educated than previous cohorts, organizations became accustomed to sustaining older workers’ employability and productivity.

Second, organizations reacted to policy reforms which dissolved government-subsidized early exit options in the light of demographic challenges. Like many other countries, the Netherlands cut early retirement opportunities and increased the statutory retirement age. A need to remain longer in employment increased the return on investment period for human capital investments and had a positive influence on older workers’ participation in development (Montizaan, Cörvers, & De Grip, 2010).

Third, workers’ retention and development became crucial to organizations’ stability and competitiveness in the quickly changing economic and technological context (Armstrong-Stassen & Ursel, 2009; Barabasch et al., 2012), a conclusion corroborated by qualitative research (Conen, Henkens, & Schippers, 2014; Moen et al., 2017). Conen and colleagues (2014) argue that the debate regarding pension reforms made employers realize that the extension of working lives was unavoidable. New cohorts entered older age with different attitudes toward work, and tight labor markets provided them a stronger position with which to negotiate job requirements, such as opportunities for continuous development (Cascio, 2019). Additionally, workplaces were becoming more older-age friendly, with progress toward counteracting age discrimination (Lahey, 2010).

Fourth was an improving economic situation. The first wave of this study was conducted during an economic slowdown when participation in adult education of older groups was low (Munnell & Rutledge, 2013). During crises, demand for work decreases and enterprises consequently cut costs, which stimulates policies focused on exit rather than retention of older workers (Armstrong-Stassen & Cattaneo, 2010).

Some limitations of the current study should be acknowledged. Although we selected several HR practices that demonstrate organizational approaches to age management, other research assesses broader lists of such practices (Kooij et al., 2014; Van Dalen et al., 2015). Although the longitudinal perspective used in this study was unique, we were unable to use a panel approach and trace changes at the organizational level, instead relying on population-averaged results. We do not control for some potentially relevant organizational characteristics, such as the average worker’s tenure and the percentage of new hires in organizations. Additionally, we only studied employers in the Netherlands. Though we believe that changes in the Dutch pension system restricting early retirement have boosted employer policies for the retention of older workers, we lack comparative data on changes in organizational policies in other countries that refrained from implementing these types of reforms.

Most Western countries are developing policies to deal with the ongoing aging of their population. Concerns regarding the long-term sustainability of the welfare state have urged national governments to redesign their pension systems. Most significant reforms include higher retirement ages and curtailing benefit levels. The reforms nudge people to delay their retirement or to return to the labor market after retirement. This study is among the first to show that employers have been changing their approach to older workers in the past decade in a country that is seen as a forerunner in increasing retirement ages. More involvement of employers in retaining older adults in the workplace may signal a growing awareness of a changing demographic reality.

## Funding

This work has received founding from the European Union’s Horizon 2020 research and innovation program under the Marie Skłodowska-Curie grant agreement No 748671 – LEEP – H2020-MSCA-IF-2016/H2020-MSCA-IF-2016. This work was also supported by the Netherlands Organization for Scientific Research (NWO) with the VICI Grant 453-14-001 to K. Henkens and by the Network for Studies on Pensions, Aging and Retirement (Netspar).

## Conflict of Interest

None reported.

## Supplementary Material

gnaa037_suppl_Supplementary_Appendix
