# Supplementary material for: The Proactive Shift in Managing an Older Workforce 2009–2017: A Latent Class Analysis of Organizational Policies
Source: Gerontologist. 2020 May 4;60(8):1515–26. doi: 10.1093/geront/gnaa037 (PMC7681210; doi:10.1093/geront/gnaa037)
Supplement: gnaa037_suppl_Supplementary_Appendix [file gnaa037_suppl_supplementary_appendix.docx]

**Appendix**

Table A1: Results of the multinomial logistic regression on predicted membership separated by year (logit coefficients). Reference category is class “Exit”

|  | **2009** | | | **2017** | | |
| --- | --- | --- | --- | --- | --- | --- |
|  | Active | All | None | Active | All | None |
| Sector (Ref= Industrial) |  |  |  |  |  |  |
| Services | 0.55 | 0.46 | 1.05* | 2.05*** | 0.84 | 1.68** |
| Public | -0.36 | 0.43 | -0.37 | 1.11 | -0.07 | 0.80 |
| Size (Ref= 1-49) |  |  |  |  |  |  |
| 50–249 | -0.43 | 0.20 | -1.67*** | -0.49 | 1.08* | -1.26** |
| 250+ | -0.80* | 1.39*** | -2.36*** | -1.14* | 1.14* | -1.94*** |
| Knowledge intensity | -0.13 | 0.11 | 0.10 | 0.45 | -0.05 | -0.41 |
| Requires regular training | 0.34 | 0.52 | -0.39 | 0.22 | 0.83 | -0.23 |
| Experienced shortages | 0.57 | 0.09 | 0.13 | 0.59 | 0.01 | 0.22 |
| Strong role of labor unions | -0.64 | -0.29 | -1.44*** | -0.28 | 0.15 | -0.66 |
| Perc. older (Ref.=0-9) |  |  |  |  |  |  |
| 10–19 | -0.54 | 0.42 | -0.93* | 0.96 | 0.54 | 0.17 |
| 20–29 | -0.56 | 0.73 | -1.25** | -0.54 | -0.94 | -1.75* |
| 30–39 | -1.72** | 0.23 | -1.52** | -0.78 | -0.07 | -2.33** |
| 40–49 | -1.86** | 0.94 | -2.11*** | -0.93 | 0.12 | -2.50* |
| 50–59 | -1.29 | 0.77 | -1.89** | -0.14 | 1.07 | -0.65 |
| 60–100 | -1.48 | 0.19 | -0.94 | -0.77 | 0.30 | -1.70 |
| Perc . women (Ref=0-9) |  |  |  |  |  |  |
| 10–19 | 1.15* | -0.14 | 0.81 | 0.49 | 0.11 | 0.50 |
| 20–39 | 1.62** | -0.10 | 1.62** | 1.93** | 0.65 | 2.14** |
| 40–59 | 2.05*** | 0.97 | 2.26*** | 0.76 | 0.16 | 0.64 |
| 60–79 | 2.27*** | 0.79 | 1.71** | 0.74 | -0.21 | 0.66 |
| 80–100 | 0.98 | -0.47 | 1.59** | 2.04 | 1.65 | 1.67 |
| Constant | -1.08 | -2.02** | 1.08* | 0.69 | -0.31 | 2.75** |

*Note*. 2009 = 1,058; 2017 = 1,273; MI (imputations with 50 m; 185 missing values imputed).
*p < .05. **p < .01. p < .001.

Models are performed separately for both waves. Coefficients are relative to the reference group (“Exit”). For binary predictors, coefficients indicate multinomial logit estimate comparing both categories of the predictor (a one unit increase) for the class relative to the reference group, with other variables held constant. For example, in the model for 2017 the multinomial logit for service sector relative to industry sector is 2.05 unit higher for being in “Active” class relative to “Exit” class (significant a p<001), with all other predictors held constant. For the 2009 model, the multinomial logit for size group 50-249 compared to 1-49 is 0.43 unit lower for being in “Active” class (not significant), 0.2 higher for being in “All” class (not significant) and 1.67 lower for being in “None” class (significant a p<.001), relative to “Exit” class. Substantial interpretation of the results is more straightforward in the form of predicted probabilities for cluster membership, as shown in Table 4 in the main text.

*Figure A1:* Sensitivity analysis: Comparison of the crisp based on probability of membership to each class and categorical based on the most probable class membership solution.
